# Initial Imaging for Adults With Maxillofacial Trauma in a National Claims Database

**DOI:** 10.1001/jamanetworkopen.2025.58293

**Published:** 2026-02-17

**Authors:** Gordon C. Wong, Yao Song, Robert D. Sampson, Lu Wang, Kevin C. Chung

**Affiliations:** 1 Section of Plastic Surgery, Department of Surgery, University of Michigan Medical School, Ann Arbor; 2 Department of Biostatistics, School of Public Health, University of Michigan, Ann Arbor; 3 Section of Plastic Surgery, Department of Surgery, Michigan Medicine, Ann Arbor

## Abstract

**Importance:**

Plain radiography offers limited diagnostic value in maxillofacial trauma and is considered low-value care. Yet, when imaging is indicated, plain radiography remains commonly used despite guidelines favoring computed tomography (CT).

**Objective:**

To examine national trends in, factors associated with, and consequences of low-value plain radiography use in the initial imaging of adults with maxillofacial trauma.

**Design, Setting, and Participants:**

This retrospective cohort study used a population-based sample of administrative claims data from the Merative MarketScan Commercial Claims and Encounters Database from January 1, 2013, to December 31, 2022. Participants were adults aged 18 years or older with an *International Classification of Diseases, Ninth Revision (ICD-9)* or* International Statistical Classification of Diseases and Related Health Problems, Tenth Revision (ICD-10)* diagnosis of facial trauma who received either plain radiography (nose, face, or orbit) or CT of the face or orbit within 7 days of diagnosis. Individuals with mandibular trauma, mandibular imaging, or prior facial imaging were excluded. Data were analyzed from December 2024 to June 2025.

**Exposure:**

Initial imaging modality for facial trauma: plain radiography (nose, face, or orbit) or CT (face or orbit).

**Main Outcomes and Measures:**

The primary outcome was prevalence of low-value (plain radiography) imaging. Secondary outcomes included patient and practitioner characteristics associated with radiography use and downstream outcomes, including follow-up CT, diagnostic delays, and imaging-related costs. Multivariable logistic regression identified factors associated with low-value imaging.

**Results:**

Among 281 421 patients (mean [SD] age, 38.9 [15.0] years; 51.6% female), 72 125 (25.6%) received low-value plain radiography as their initial imaging. Plain radiography use declined from 32.9% of cases in 2013 to 18.0% in 2022. Female vs male sex (odds ratio [OR], 1.50; 95% CI, 1.47-1.54), being seen in an urgent care vs office-based setting (OR, 1.76; 95% CI, 1.67-1.87), and being seen by a family medicine practitioner vs an internist (OR, 1.43; 95% CI, 1.33-1.54) were associated with low-value imaging. In contrast, being seen by a surgical specialist vs internist (OR, 0.09; 95% CI, 0.08-0.11) or in an emergency department vs office setting (OR, 0.07; 95% CI, 0.07-0.07) were associated with significantly lower odds of receiving low-value imaging. In all, 5.5% of radiograph recipients underwent follow-up CT within 7 days, and 7.6% experienced a diagnostic delay. Although median total costs were lower for plain radiography ($56 [IQR, $29-$139]) than CT ($378 [IQR, $89-$845]), radiograph vs CT represented a greater proportion of total (mean [SD], 26% [24%] vs 21% [21%]) and out-of-pocket (mean [SD], 22% [30%] vs 15% [24%]) costs in low-acuity settings.

**Conclusions and Relevance:**

In this cohort study of adults with maxillofacial trauma, low-value plain radiography was frequently used despite clear guidelines favoring CT. Targeted interventions to reduce low-value imaging may improve diagnostic accuracy, reduce unnecessary costs, and advance value-based care.

## Key Points

**Question:**

What are the prevalence and impact of low-value plain radiography use for initial imaging in adults with maxillofacial trauma?

**Findings:**

In this cohort study of 281 421 patients with maxillofacial trauma, 26% received low-value plain radiography as their initial imaging despite guideline recommendations favoring computed tomography (CT). Among patients receiving plain radiography, 6% required follow-up CT and 8% experienced delayed fracture diagnosis.

**Meaning:**

Despite guideline recommendations favoring CT, low-value plain radiography remained common in facial trauma evaluation, highlighting opportunities to improve adherence to evidence-based imaging practices.

## Teaser

This cohort study examines prevalence of and factors associated with use of low-value plain radiography vs guideline-recommended computed tomography as the initial imaging for adults with maxillofacial trauma.

## Introduction

Facial trauma is one of the most common reasons for emergency department visits, with its incidence increasing over the years.^1,2,3^ Accurate and timely diagnosis is essential to ensuring optimal outcomes and preventing complications.^4,5^ Although computed tomography (CT) has become the diagnostic standard for evaluating maxillofacial fractures because of its superior sensitivity and specificity, plain radiographs of the face, nose, and orbits continue to be frequently ordered in clinical practice.^6,7,8,9^ These low-sensitivity imaging studies offer limited diagnostic value and often fail to detect clinically significant injuries that would otherwise be identified by CT.^10^ In recognition of these limitations, both the American College of Radiology (ACR) and the American Society of Plastic Surgeons recommend against the routine use of most plain radiography in maxillofacial trauma evaluation.^11,12,13^

Despite these guidelines, plain radiography remains in use, representing low-value care interventions that provide minimal clinical benefit relative to their cost. These studies expose patients to unnecessary radiation, delay definitive diagnosis, and contribute to avoidable health care expenditure. As health systems increasingly embrace value-based care, reducing low-value imaging is critical to improving efficiency and patient outcomes.

To date, the national prevalence, drivers, and downstream consequences of plain radiography use in maxillofacial trauma have not been well characterized. By identifying patterns of plain radiography use, examining associated demographic factors, and assessing downstream consequences, this study aimed to inform efforts to reduce low-value care for maxillofacial trauma and promote evidence-based imaging practices. We hypothesized that plain radiography has been increasingly supplanted by CT as the initial imaging modality in maxillofacial trauma.

## Methods

We conducted a retrospective cohort study using data from the Merative MarketScan Commercial Claims and Encounters Database from January 1, 2013, to December 31, 2022. MarketScan is a large nationwide administrative claims database that captures longitudinal, deidentified health care utilization data from over 273 million patients in the US, making it suitable for evaluating trends in clinical practice. The University of Michigan institutional review board deemed this study exempt, and patient consent was not required because data were deidentified in compliance with the Health Insurance Portability and Accountability Act. This study adhered to the Strengthening the Reporting of Observational Studies in Epidemiology (STROBE) reporting guideline.

Using codes from the *International Classification of Diseases, Ninth Revision (ICD-9)* (from 2013-2016) or *International Statistical Classification of Diseases and Related Health Problems, Tenth Revision (ICD-10)* (from 2016-2022) as well as *Current Procedural Terminology (CPT)* codes (eTables 1 and 2 in Supplement 1), we identified adult patients (aged ≥18 years) diagnosed with maxillofacial trauma (excluding mandibular trauma) who underwent either CT of the maxillofacial region or orbits or plain radiography of the face, nose, or orbits within 7 days following the initial trauma diagnosis. These imaging modalities were selected based on ACR Appropriateness Criteria for acute facial trauma, which provide consensus-based recommendations on imaging utility.^12,13^ Of importance, the ACR guidelines do not address the decision of which patients warrant imaging. Instead, they provide recommendations on the appropriate imaging modality once clinical assessment has determined that imaging is necessary. Maxillofacial and orbital CT scans were classified as high-value imaging because of their established diagnostic utility.^10^ In contrast, plain radiography of the face, nose, and orbits was deemed low value, as it is considered “usually not appropriate” for initial evaluation of facial trauma.^13^ We excluded individuals with prior facial imaging and those receiving other imaging types, such as sinus radiographs, mandibular radiographs, and head CT, as the appropriateness of these imaging types is context dependent and cannot be reliably assessed using administrative data that lack clinical detail. The 7-day window was chosen to capture imaging plausibly related to the index facial trauma while accommodating delays in care across diverse health care settings. Because some patients may have received an initial fracture diagnosis based on clinical assessment alone, with imaging delayed by several days due to access limitations, we included all imaging performed within 7 days following the diagnosis date. For patients who received multiple imaging studies, we analyzed only the first modality obtained to assess initial imaging decisions.

Patients were categorized based on their initial imaging modality. Those who received plain radiography first were classified as receiving low-value imaging, whereas those who received CT first were classified as receiving high-value imaging. When plain radiography and CT were performed on the same day, we assumed the plain radiography preceded CT, reflecting typical clinical workflows. Among radiograph recipients, we also identified the specific radiograph type (facial, nasal, orbital, or multiple).

### Outcomes

The primary outcome was the incidence of high-value vs low-value imaging. Secondary outcomes included patient- and encounter-level factors associated with low-value imaging, as well as downstream consequences such as follow-up CT, diagnostic delays, and imaging-related costs. We performed multivariable logistic regression to identify factors associated with receiving plain radiography vs CT as the initial imaging modality. Covariates included age, sex, geographic region, insurance plan type (eg, high-deductible health plan), place of service (emergency department, urgent care, or outpatient clinic), practitioner specialty, and rurality. Rurality was inferred from the metropolitan statistical area (MSA) designation of each patient’s home address, with claims lacking MSA codes being considered rural.^14^ To evaluate downstream consequences of low-value imaging, we analyzed (1) the proportion of patients who underwent a follow-up CT within 7 days after plain radiography imaging, (2) total and out-of-pocket costs for radiography, and (3) the diagnostic value of the plain radiography and potential diagnostic delays. Costs of radiographs were derived from claims data using relevant *CPT* codes, with extreme outliers (defined as values exceeding 3 SDs above the mean) adjusted to the threshold value to minimize their distorting effect. To assess diagnostic delays in the radiography group, we identified patients whose initial diagnosis did not indicate fracture (eg, contusions, abrasions, open wounds, or lacerations) but who subsequently received a fracture diagnosis more than 3 days after initial imaging. This subgroup analysis allowed us to quantify potential missed or delayed fracture diagnoses associated with low-value imaging.

### Statistical Analysis

Data were extracted and analyzed from December 2024 to June 2025 using SAS software, version 9.3 (SAS Institute Inc). All statistical analysis was performed using R, version 4.2.3 (R Project for Statistical Computing). Statistical significance was set at 2-sided *P* < .05. Continuous variables were summarized as mean and SD, while categorical variables were presented as counts and percentages. Baseline characteristics were compared using χ^2^ tests for categorical variables and 2-sample *t* tests for continuous variables. The Cochran-Armitage test was applied to analyze the change in the proportion of CT scans and radiographs performed over time. Cost data were presented as median with IQR. Multivariable logistic regression was used to identify factors that were associated with receipt of plain radiography as the initial imaging modality. To evaluate potential collinearity among covariates, we examined pairwise associations using Pearson correlations for continuous variables, Cramér V for categorical variables, and correlation ratios for continuous-categorical variable pairs.

## Results

From 2013 to 2022, we identified 281 421 patients with facial trauma aged 18 years or older who underwent imaging within 7 days of diagnosis. Table 1 details cohort demographics. The mean (SD) age was 38.9 (15.0) years; 145 319 patients (51.6%) were female and 136 102 (48.4%) were male. Most patients resided in urban areas (224 224 [79.7%]), were treated in the South vs other US regions (126 342 [44.9%]), and were enrolled in low-deductible health plans (222 434 [79.0%]). Hospital outpatient departments (108 928 [38.7%]) and emergency departments (103 667 [36.8%]) were the most common sites of care. Emergency medicine physicians (27 971 encounters [9.9%]) and family medicine physicians (15 945 encounters [5.7%]) were the most frequently identified practitioners.

**Table 1.** Cohort Demographics

Characteristic	Patients, No. (%)			*P* value
	Total (N = 281 421)	Computed tomography (n = 209 296)	Plain radiography (n = 72 125)	
Age, mean (SD), y	38.9 (15.0)	39.3 (15.0)	37.8 (14.8)	<.001
Sex				
Female	145 319 (51.6)	102 459 (49.0)	42 860 (59.4)	<.001
Male	136 102 (48.4)	106 837 (51.0)	29 265 (40.6)	
Region				
Northeast	52 435 (18.6)	37 768 (18.0)	14 667 (20.3)	<.001
North Central	59 757 (21.2)	44 695 (21.4)	15 062 (20.9)	
South	126 342 (44.9)	96 244 (46.0)	30 098 (41.7)	
West	40 233 (14.3)	28 838 (13.8)	11 395 (15.8)	
Unknown	2654 (0.9)	1751 (0.8)	903 (1.3)	
Urban				
Yes	224 224 (79.7)	165 837 (79.2)	58 387 (81.0)	<.001
No	34 561 (12.3)	26 384 (12.6)	8177 (11.3)	
Unknown	22 636 (8.0)	17 075 (8.2)	5561 (7.7)	
Site of service				
Office	38 786 (13.8)	12 912 (6.2)	25 874 (35.9)	<.001
Urgent care	9868 (3.5)	2075 (1.0)	7793 (10.8)	
Outpatient hospital	108 928 (38.7)	85 895 (41.0)	23 033 (31.9)	
Emergency department	103 667 (36.8)	90 636 (43.3)	13 031 (18.1)	
Other^a^	20 172 (7.2)	17 778 (8.5)	2394 (3.3)	
Practitioner type				
Internal medicine	6062 (2.2)	3059 (1.5)	3003 (4.2)	<.001
Emergency medicine	27 971 (9.9)	22 445 (10.7)	5526 (7.7)	
Family medicine	15 945 (5.7)	5535 (2.6)	10 410 (14.4)	
Ophthalmology	1464 (0.5)	1273 (0.6)	191 (0.3)	
ENT	2300 (0.8)	1779 (0.8)	521 (0.7)	
Plastic surgery	880 (0.3)	728 (0.3)	152 (0.2)	
Other^b^	226 799 (80.6)	174 477 (83.3)	52 322 (72.5)	
Insurance plan type				
High deductible	50 956 (18.1)	38 341 (18.3)	12 615 (17.5)	<.001
Low deductible	222 434 (79.0)	165 019 (78.8)	57 415 (79.6)	
Unknown or none	8031 (2.9)	5936 (2.8)	2095 (2.9)	

Abbreviation: ENT, ear, nose, and throat.

^a^ Included settings that were not clinically relevant, such as billing and administrative classifications.

^b^ Included nonspecific practitioner codes, such as multispecialty physician groups or specialty care facilities.

Overall, 72 125 patients (25.6%) received low-value imaging, whereas 209 296 (74.4%) received high-value imaging (Table 2). Among the plain radiography group, nasal radiography was most common (42 746 patients [59.3%]), followed by facial (21 100 [29.3%]) and orbital (5048 [7.0%]) radiography. Compared with the CT group, patients in the radiography group were younger on average (mean [SD] age, 37.8 [14.8] vs 39.3 [15.0] years; *P* < .001), were more likely to be female (42 860 [59.4%] vs 102 459 [49.0%]; *P* < .001), and more often received imaging in outpatient offices (25 874 [35.9%] vs 12 912 [6.2%]) or urgent care centers (7793 [10.8%] vs 2075 [1.0%]). In contrast, patients in the CT group were more frequently evaluated in hospital outpatient settings (85 895 [41.0%] vs 23 033 [31.9%]) or emergency departments (90 636 [43.3%] vs 13 031 [18.1%]). Family medicine physicians were the practitioners most likely to order plain radiography (10 410 [14.4%]), whereas emergency medicine physicians predominated in the CT group (22 445 [10.7%]).

**Table 2.** Types of Imaging Modality Use in Patients With Maxillofacial Trauma by Year^a^

Year	All imaging, No.	Computed tomography, No. (% of all imaging)	Plain radiography				
			Total, No. (% of all imaging)	Subtypes, No. (% of total plain radiography)			
				Facial	Nasal	Orbit	Multiple
2013	35 477	23 788 (67.1)	11 689 (32.9)	3043 (26.0)	7114 (60.8)	881 (7.5)	651 (5.6)
2014	38 996	26 945 (69.1)	12 051 (30.9)	3129 (26.0)	7363 (61.1)	931 (7.7)	628 (5.2)
2015	26 016	18 583 (71.4)	7433 (28.6)	2029 (27.3)	4485 (60.3)	576 (7.8)	343 (4.6)
2016	32 279	23 766 (73.6)	8513 (26.4)	2667 (31.3)	4850 (57.0)	621 (7.3)	375 (4.4)
2017	30 677	22 990 (74.9)	7687 (25.1)	2466 (32.1)	4396 (57.2)	520 (6.8)	305 (4.0)
2018	30 828	23 536 (76.3)	7292 (23.7)	2204 (30.2)	4292 (58.9)	490 (6.7)	306 (4.2)
2019	24 831	19 256 (77.5)	5575 (22.5)	1708 (30.6)	3283 (58.9)	370 (6.6)	214 (3.8)
2020	19 111	15 333 (80.2)	3778 (19.8)	1245 (33.0)	2170 (57.4)	230 (6.1)	133 (3.5)
2021	21 460	17 264 (80.4)	4196 (19.6)	1323 (31.5)	2513 (59.9)	224 (5.3)	136 (3.2)
2022	21 746	17 835 (82.0)	3911 (18.0)	1286 (32.9)	2280 (58.3)	205 (5.2)	140 (3.6)
Total	281 421	209 296 (74.4)	72 125 (26.6)	21 100 (29.3)	42 746 (59.3)	5048 (7.0)	3231 (3.6)

^a^ *P* < .001 for all comparisons. All *P *values were from Cochran-Armitage tests with year as the ordered score.

Low-value imaging declined steadily over the study period, from 11 689 of 35 477 cases (32.9%) in 2013 to 3911 of 21 746 cases (18.0%) in 2022 (*P* < .001) (Figure). This decline was modality specific, with orbital radiography showing the largest reduction in examinations (from 881 to 205 [76.7% decrease]), followed by nasal (from 7114 to 2280 [68.0% decrease]) and facial (from 3043 to 1286 [57.7% decrease]) radiography. Concurrently, the annual rate of CT as the initial modality rose from 23 788 of 35 477 cases (67.1%) in 2013 to 17 835 of 21 746 (82.0%) in 2022 (*P* < .001) (Table 2). Among the plain radiography group, 3965 of 72 125 patients (5.5%) received follow-up CT imaging within 7 days, with the highest rates among those who had facial or multiple types of radiography (eTable 3 in Supplement 1). Of the 46 166 patients who received plain radiography without an initial fracture diagnosis, 11 155 (24.2%) were subsequently diagnosed with a fracture, and 850 of those (7.6%) had a fracture diagnosis delayed by more than 3 days (eTable 4 in Supplement 1). This overall rate was largely driven by nasal radiographs, which had a 41.4% yield for new fracture diagnoses (8718 of 21 042 films), compared with 1563 of 18 233 (8.6%) for facial radiographs, 179 of 4796 (3.7%) for orbital radiographs, and 695 of 2095 (33.2%) for multiple radiograph types. The high yield for nasal films may reflect the frequency of isolated nasal trauma and initial diagnostic uncertainty.

**Figure.**
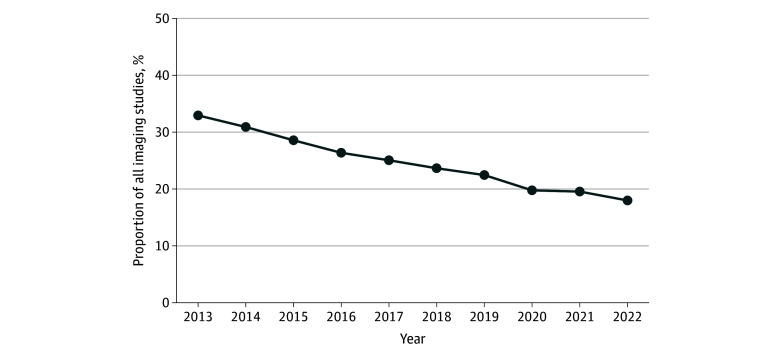
Plain Radiography Use by Year in Patients With Maxillofacial Trauma

For low-value imaging episodes, the median total cost was substantially higher for patients who received CT compared with those who received plain radiography ($378 [IQR, $89-$845] vs $56 [IQR, $29-$139]) (Table 3). Out-of-pocket costs followed a similar pattern, with a median of $19 (IQR, $0-$132) for CT and $8 (IQR, $0-$46) for radiographs. However, despite the lower absolute cost, plain radiography represented a greater share of both total and out-of-pocket costs within the encounter. On average, radiographs accounted for a mean (SD) of 26% (24%) of the total encounter cost and 22% (30%) of the out-of-pocket cost, compared with 21% (21%) and 15% (24%) for CT, respectively. This likely reflects differences in the care settings where these imaging modalities are used: CT is more frequently performed in higher-acuity environments such as emergency departments or hospital outpatient settings, where the overall cost of care is higher. In contrast, plain radiography images are often obtained in lower-cost settings such as urgent care centers or outpatient clinics, where they represent a larger proportion of the total expenditure.

**Table 3.** Comparative Costs and Cost Sharing by Imaging Type

Cost	Computed tomography	Plain radiography
Total, median (IQR), $	378 (89-845)	56 (29-139)
Out of pocket, median (IQR), $	19 (0-132)	8 (0-46)
Proportion of total encounter cost, mean (SD), %	21 (21)	26 (24)
Proportion of out-of-pocket cost, mean (SD), %	15 (24)	22 (30)

Multivariable logistic regression identified several significant factors associated with low-value imaging use (Table 4). Each year of the study period was associated with a decrease in odds of radiography-first imaging (OR per year increase, 0.91; 95% CI, 0.90-0.91; *P* < .001). Increasing age was associated with a slight but statistically significant decrease in odds of receiving plain radiography (OR per year increase, 0.99; 95% CI, 0.99-0.99; *P* < .001). Female patients were more likely to receive low-value imaging compared with males (OR, 1.50; 95% CI, 1.47-1.54; *P* < .001). Geographic variation was notable; compared with the Northeast as the reference category, patients in the South had the lowest likelihood of receiving plain radiography (OR, 0.82; 95% CI, 0.80-0.84; *P* < .001), followed by the West (OR, 0.89; 95% CI, 0.86-0.92; *P* < .001) and the North Central region (OR, 0.90, 95% CI, 0.87-0.92; *P* < .001). Urban residence was associated with modestly decreased odds of plain radiography (OR, 0.94; 95% CI, 0.92-0.97; *P* < .001) compared with rural settings. Clinical setting was also associated with imaging modality choice. Compared with office-based encounters, emergency department visits were associated with considerably reduced odds of radiography use (OR, 0.07; 95% CI, 0.07-0.07; *P* < .001), suggesting a strong preference for CT in emergency settings. Similarly, outpatient hospital visits showed substantially lower radiography use (OR, 0.12; 95% CI, 0.12-0.13; *P* < .001). In contrast, urgent care settings demonstrated higher odds of plain radiography use compared with office-based settings (OR, 1.76; 95% CI, 1.67-1.87; *P* < .001).

**Table 4.** Multivariable Logistic Regression of Factors Associated With Low-Value Imaging Use in Facial Trauma

Characteristic	OR (95% CI)	*P* value
Study year, per 1-y increase	0.91 (0.90-0.91)	<.001
Age, per 1-y increase	0.99 (0.99-0.99)	<.001
Sex		
Female	1.50 (1.47-1.54)	<.001
Male	1 [Reference]	NA
Rural status		
Rural	1 [Reference]	NA
Urban	0.94 (0.92-0.97)	<.001
Unknown	0.99 (0.96-1.06)	.73
Region		
Northeast	1 [Reference]	NA
North Central	0.90 (0.87-0.92)	<.001
South	0.82 (0.80-0.84)	<.001
West	0.89 (0.86-0.92)	<.001
Unknown	0.94 (0.85-1.04)	.25
Insurance plan type		
Non-HDHP	1 [Reference]	NA
HDHP	1.02 (1.00-1.05)	.09
Unknown or none	1.02 (0.96-1.09)	.54
Site of service		
Office	1 [Reference]	NA
Urgent care	1.76 (1.67-1.87)	<.001
Outpatient hospital	0.12 (0.12-0.13)	<.001
Emergency department	0.07 (0.07-0.07)	<.001
Other^a^	0.07 (0.06-0.07)	<.001
Practitioner specialty		
Internal medicine	1 [Reference]	NA
Emergency medicine	0.86 (0.80-0.92)	<.001
Family medicine	1.43 (1.33-1.54)	<.001
Ophthalmology	0.09 (0.08-0.11)	<.001
Otolaryngology	0.13 (0.11-0.14)	<.001
Plastic surgery	0.24 (0.19-0.29)	<.001
Other^b^	0.90 (0.85-0.96)	.002

Abbreviations: HDHP, high-deductible health plan; NA, not applicable; OR, odds ratio.

^a^ Included settings that were not clinically relevant, such as billing and administrative classifications.

^b^ Included nonspecific practitioner codes, such as multispecialty physician groups or specialty care facilities.

Practitioner specialty was also associated with imaging decisions. Family medicine practitioners had higher odds of ordering plain radiography films compared with internists (OR, 1.43; 95% CI, 1.33-1.54; *P* < .001). Surgical specialists showed particularly strong preferences for CT: ophthalmologists (OR, 0.09; 95% CI, 0.08-0.11; *P* < .001), otolaryngologists (OR, 0.13; 95% CI, 0.11-0.14; *P* < .001), and plastic surgeons (OR, 0.24; 95% CI, 0.19-0.29; *P* < .001) were all significantly less likely to order radiographs compared with internists. Emergency medicine practitioners showed modestly reduced radiography use compared with internists (OR, 0.86; 95% CI, 0.80-0.92; *P* < .001). Insurance type was not significantly associated with receiving plain radiography over CT. Correlation analysis did not indicate any association between any of the covariates (eFigure in Supplement 1).

## Discussion

In this national claims-based analysis of over 280 000 adults with facial trauma, 1 in 4 patients received low-value plain radiography as their initial imaging study, despite long-standing clinical guidelines recommending CT as the preferred modality.^11,12,13^ Although plain radiography use declined by nearly half over the 9-year study period, its continued use had measurable consequences, with 5.5% of plain radiography recipients requiring subsequent CT and 7.6% of patients without an initial fracture diagnosis experiencing a diagnostic delay of more than 3 days. Women were 50% more likely than men to receive plain radiography, and urgent care settings had 76% higher radiography use than office-based settings. These findings underscore a persistent gap between evidence-based practice and actual clinical imaging patterns in facial trauma care.

The observed decline in plain radiography represents progress toward more evidence-based, value-driven care. This shift likely reflects increased access to CT imaging and broader dissemination of guidelines discouraging plain radiography. Over the past decade, CT utilization has risen steadily across clinical settings, in part due to improvements in availability and efficiency.^15,16,17^ Concurrently, specialty societies in radiology and plastic surgery have issued clear recommendations favoring CT over plain radiography for evaluating facial fractures.^11,12,13^ Despite these improvements, a substantial proportion of patients still receive low-value plain radiography as their initial imaging, indicating only partial adoption of guidelines. CT is unequivocally the diagnostic modality of choice in maxillofacial trauma, given its superior sensitivity, specificity, and ability to guide treatment decisions.^10,18,19^ Factors such as injury severity, mechanism, patient characteristics, and physical examination findings should appropriately guide the decision of whether imaging is needed, but once imaging is indicated, current guidelines and expert reviews consistently recommend CT as the preferred modality.^20,21^ To further reduce the use of low-yield imaging, multifaceted interventions, including clinical decision support, targeted practitioner education, and expanded access to CT in lower-acuity settings, can help align diagnostic practices with current evidence and improve the value of care delivery.

The use of plain radiography in maxillofacial trauma carries tangible downstream consequences. Although some patients who receive an initial radiograph are ultimately diagnosed with a fracture without additional imaging, this subgroup should be interpreted cautiously. The main issue with plain radiography is its limited sensitivity, with up to 12% of facial fractures missed compared with CT.^22^ False-negative results can delay diagnosis, prompt repeat visits, and fragment care, ultimately impacting surgical planning, pain management, and eventual functional and aesthetic outcomes.^4,5^ The 5.5% rate of subsequent CT among patients initially receiving radiographs in our cohort highlights the inefficiency of starting with a low-sensitivity test when clinical suspicion for fracture is present. Moreover, many patients diagnosed on radiograph still require specialist follow-up or operative planning and will ultimately undergo CT. As our study captured only the first imaging study within 7 days of diagnosis, we inevitably underestimated the true proportion of patients initially receiving radiographs who later proceeded to CT. Financially, the cumulative cost of low-value imaging is substantial. Although the median cost of radiography for an individual patient in our dataset was $56, this figure likely underestimates the true economic burden, as it excludes professional interpretation fees, staff time, and institutional overhead. Given the high prevalence of plain radiography in this cohort, even modest per-patient costs translate into meaningful health care expenditures. Moreover, these estimates do not capture indirect and intangible costs associated with diagnostic delays, missed injuries, or patient anxiety. The true economic and clinical impact is therefore likely to be substantially higher. Efforts to curtail unnecessary imaging in facial trauma are therefore warranted and represent a meaningful opportunity to improve both the quality and value of care.

Imaging modality selection reflects a complex interplay of patient, practitioner, and systemic factors. We found that women were 50% more likely than men to receive low-value imaging. Although the exact drivers remain unclear, several potential contributors warrant consideration, including the underestimation of injury severity in women, differences in pain expression or patient advocacy, and implicit biases in clinical decision-making. Implicit gender bias in health care is well documented, including underestimation of pain and disparities in triage.^23,24,25,26^ Previous work by Gomez et al^27^ found that women with traumatic injuries were less likely than men to be triaged to trauma centers despite similar injury severity, which, in the context of facial trauma, may funnel women toward settings where low-value imaging is more prevalent. Although unmeasured confounding, such as injury mechanism and severity, cannot be excluded, the alignment of our findings with broader evidence on gender disparities suggests bias may influence even acute diagnostic decisions.^28,29,30,31,32^ Such disparities risk tangible consequences such as delayed or missed diagnoses, additional imaging, increased out-of-pocket costs, and potential harm from fragmented care. For a condition where accurate and timely diagnosis is crucial to avoiding poor functional and aesthetic outcomes, sex-based differences in diagnostic imaging quality underscore the urgent need to bridge equity gaps. These findings call for greater scrutiny of how implicit bias may influence diagnostic pathways and reinforce the need for standardized, guideline-driven imaging protocols that reduce variability and promote equitable care regardless of gender.

Apart from patient-level factors, both treatment setting and practitioner specialty were independently associated with the likelihood of receiving low-value imaging. Patients evaluated in office or urgent care settings were significantly more likely to receive plain radiography than those seen in emergency departments or outpatient hospitals. Similarly, being seen by generalist practitioners, particularly family medicine and internal medicine physicians, was associated with higher odds of low-value imaging, while surgical specialists were far less likely to rely on plain films. These patterns likely reflect a combination of resource availability, injury severity, and clinical norms. Offices and urgent care clinics often lack on-site CT capability and may default to plain films out of convenience or logistic constraints. Additionally, patients with more severe trauma are more likely to be seen in emergency departments or be referred to specialists, which may contribute to differences in imaging choices. Surgical specialists may also favor CT for its diagnostic accuracy and utility in preoperative planning. Although resource limitations in lower-acuity settings may justify some use of plain radiography, its diagnostic limitations in facial trauma are well established. This creates a 2-tiered system in which imaging and care quality may depend more on where patients seek care and who evaluates them than on clinical factors. To address this, targeted interventions are needed: streamlined CT referral pathways for urgent care settings, embedded decision-support tools in electronic health record systems, and cross-specialty education on facial trauma imaging standards. Together, these highlight an opportunity to improve diagnostic equity by expanding infrastructure, training, and support for generalists and lower-resourced care environments, ensuring that all patients, regardless of where and by whom they are seen, receive timely, appropriate, and guideline-concordant imaging.

### Limitations

Our study has several limitations inherent to the use of administrative data. First, the lack of clinical granularity, such as injury severity, physical examination findings, or physician decision-making rationale, precluded full assessment of factors influencing imaging choice. Patient-reported outcomes, such as functional recovery, cosmetic results, or complications, were also not captured, limiting our ability to assess whether delayed imaging or diagnosis adversely impacted clinical outcomes. Second, restricting imaging capture to the first 7 days after the index trauma ensured that included studies were diagnostic in intent but likely underestimated how many patients who initially received radiographs later proceeded to CT. Additionally, because claims data do not capture missed fractures, delayed diagnoses, or related complications, our study could not assess these downstream clinical and financial consequences. Third, our cohort consisted solely of commercially insured patients, which may limit generalizability to populations with other forms of insurance. Fourth, unmeasured confounders (eg, patient preferences, practitioner habits, or institutional protocols) could not be accounted for, though we adjusted for available demographic and clinical variables. Despite these constraints, our findings provide valuable insights into clinical imaging trends across diverse practice settings.

## Conclusions

In this cohort study of adult patients with maxillofacial trauma, plain radiography use gradually declined during the study period, demonstrating meaningful progress toward value-based imaging. However, our findings suggest persistent gaps in guideline adherence that disproportionately affect women and patients in low-acuity settings. As CT becomes more accessible, the adoption of guideline-concordant care must be prioritized to ensure all patients receive optimal diagnostics, regardless of gender or care setting.

## Notes

Supplement 1. **eTable 1.** Maxillofacial Trauma *International Classification of Diseases (ICD)* Codes **eTable 2.** Maxillofacial Trauma Imaging *Current Procedural Terminology (CPT)* Codes **eTable 3.** Rates of Follow-Up Computed Tomography Within 7 Days After Initial Plain Radiography **eTable 4.** Diagnostic Outcomes After Initial Plain Radiography **eFigure.** Correlation Matrix Among All Covariates

Supplement 2.
Data Sharing Statement
