# Supplementary material for: Initial Imaging for Adults With Maxillofacial Trauma in a National Claims Database
Source: JAMA Netw Open. 2026 Feb 17;9(2):e2558293. doi: 10.1001/jamanetworkopen.2025.58293 (PMC12914484; doi:10.1001/jamanetworkopen.2025.58293)
Supplement: Supplement 1. — eTable 1. Maxillofacial Trauma International Classification of Diseases (ICD) Codes eTable 2. Maxillofacial Trauma Imaging Current Procedural Terminology (CPT) Codes eTable 3. Rates of Follow-Up Computed Tomography Within 7 Days After Initial Plain Radiography eTable 4. Diagnostic Outcomes After Initial Plain Radiography eFigure. Correlation Matrix Among All Covariates [file jamanetwopen-e2558293-s001.pdf]

Supplementary Online Content

Wong GC, Song Y, Sampson RD, Wang L, Chung KC. Initial imaging for adults with maxillofacial trauma in a national claims database. *JAMA Netw Open*. 2026;9(2):e2558293. doi:10.1001/jamanetworkopen.2025.58293

eTable 1. Maxillofacial Trauma *International Classification of Diseases (ICD)* Codes

eTable 2. Maxillofacial Trauma Imaging *Current Procedural Terminology (CPT)* Codes

eTable 3. Rates of Follow-Up Computed Tomography Within 7 Days After Initial Plain Radiography

eTable 4. Diagnostic Outcomes After Initial Plain Radiography

eFigure. Correlation Matrix Among All Covariates

This supplementary material has been provided by the authors to give readers additional information about their work.

© 2026 Wong GC et al. *JAMA Network Open*.

eTable 1. Maxillofacial Trauma *International Classification of Diseases (ICD)* Codes

Codes	Description
**Fracture**	
ICD-9	
802.0	Closed fracture of nasal bones
802.1	Open fracture of nasal bones
802.4	Closed fracture of malar and maxillary bones
802.5	Open fracture of malar and maxillary bones
802.6	Closed fracture of orbital floor (blow-out)
802.7	Open fracture of orbital floor (blow-out)
802.8	Closed fracture of other facial bones
802.9	Open fracture of other facial bones
ICD-10	
S02.2XXA	Fracture of nasal bones, initial encounter for closed fracture
S02.2XXB	Fracture of nasal bones, initial encounter for open fracture
S02.30XA	Fracture of orbital floor, unspecified side, initial encounter for closed fracture
S02.30XB	Fracture of orbital floor, unspecified side, initial encounter for open fracture
S02.31XA	Fracture of orbital floor, right side, initial encounter for closed fracture
S02.31XB	Fracture of orbital floor, right side, initial encounter for open fracture
S02.32XA	Fracture of orbital floor, left side, initial encounter for closed fracture
S02.32XB	Fracture of orbital floor, left side, initial encounter for open fracture
S02.40	Fracture of zygomatic bone, unspecified
S02.400A	Fracture of zygomatic bone, unspecified, initial encounter for closed fracture
S02.400B	Fracture of zygomatic bone, unspecified, initial encounter for open fracture
S02.401A	Fracture of zygomatic bone, right side, initial encounter for closed fracture
S02.401B	Fracture of zygomatic bone, right side, initial encounter for open fracture
S02.402A	Fracture of zygomatic bone, left side, initial encounter for closed fracture
S02.402B	Fracture of zygomatic bone, left side, initial encounter for open fracture
S02.40AA	Fracture of zygomatic bone, unspecified, initial encounter for closed fracture
S02.40AB	Fracture of zygomatic bone, unspecified, initial encounter for open fracture
S02.40BA	Fracture of zygomatic bone, right side, initial encounter for closed fracture
S02.40BB	Fracture of zygomatic bone, right side, initial encounter for open fracture
S02.40CA	Fracture of zygomatic bone, left side, initial encounter for closed fracture
S02.40CB	Fracture of zygomatic bone, left side, initial encounter for open fracture
S02.40DA	Fracture of zygomatic bone, unspecified, initial encounter for closed fracture
S02.40DB	Fracture of zygomatic bone, unspecified, initial encounter for open fracture
S02.40EA	Fracture of zygomatic bone, right side, initial encounter for closed fracture
S02.40EB	Fracture of zygomatic bone, right side, initial encounter for open fracture
S02.40FA	Fracture of zygomatic bone, unspecified, initial encounter for closed fracture
S02.40FB	Fracture of zygomatic bone, unspecified, initial encounter for open fracture
S02.411A	Fracture of zygomatic bone, right side, initial encounter for closed fracture

© 2026 Wong GC et al. *JAMA Network Open*.

S02.411B	Fracture of zygomatic bone, right side, initial encounter for open fracture
S02.412A	Fracture of zygomatic bone, left side, initial encounter for closed fracture
S02.412B	Fracture of zygomatic bone, left side, initial encounter for open fracture
S02.413A	Fracture of zygomatic bone, unspecified, initial encounter for closed fracture
S02.413B	Fracture of zygomatic bone, unspecified, initial encounter for open fracture
S02.42XA	Fracture of mandible, unspecified, initial encounter for closed fracture
S02.42XB	Fracture of mandible, unspecified, initial encounter for open fracture
S02.80XA	Fracture of other specified skull and facial bones, initial encounter for closed fracture
S02.80XB	Fracture of other specified skull and facial bones, initial encounter for open fracture
S02.81XA	Fracture of other facial bones, initial encounter for closed fracture
S02.82XB	Fracture of other facial bones, initial encounter for open fracture
S02.831A	Fracture of lateral orbital wall, right side, initial encounter for closed fracture
S02.831B	Fracture of lateral orbital wall, right side, initial encounter for open fracture
S02.832A	Fracture of lateral orbital wall, left side, initial encounter for closed fracture
S02.832B	Fracture of lateral orbital wall, left side, initial encounter for open fracture
S02.839A	Fracture of lateral orbital wall, unspecified side, initial encounter for closed fracture
S02.839B	Fracture of lateral orbital wall, unspecified side, initial encounter for open fracture
S02.841A	Fracture of medial orbital wall, right side, initial encounter for closed fracture
S02.841B	Fracture of medial orbital wall, right side, initial encounter for open fracture
S02.842A	Fracture of medial orbital wall, left side, initial encounter for closed fracture
S02.842B	Fracture of medial orbital wall, left side, initial encounter for open fracture
S02.849A	Fracture of medial orbital wall, unspecified side, initial encounter for closed fracture
S02.849B	Fracture of medial orbital wall, unspecified side, initial encounter for open fracture
S02.85XA	Fracture of orbit, unspecified, initial encounter for closed fracture
S02.85XB	Fracture of orbit, unspecified, initial encounter for open fracture
S02.92XA	Fracture of other specified skull and facial bones, initial encounter for closed fracture
S02.92XB	Fracture of other specified skull and facial bones, initial encounter for open fracture
**Non-fracture**	
ICD-9	
870.0	Laceration of skin of eyelid and periocular area
870.1	Laceration of eyelid, full-thickness, not involving lacrimal passages
870.2	Laceration of eyelid involving lacrimal passages
870.3	Penetrating wound of orbit, without mention of foreign body
870.8	Other specified open wounds of ocular adnexa
870.9	Unspecified open wound of ocular adnexa

© 2026 Wong GC et al. *JAMA Network Open*.

910.0	Abrasion or friction burn of face, neck, and scalp except eye, without mention of infection
910.8	Other and unspecified superficial injury of face, neck, and scalp, without mention of infection
918.0	Superficial injury of eyelids and periocular area
920	Contusion of face, scalp, and neck except eye(s)
921.0	Black eye, not otherwise specified
921.1	Contusion of eyelids and periocular area
921.2	Contusion of orbital tissues
921.3	Contusion of eyeball
921.9	Unspecified contusion of eye
925.1	Crushing injury of face and scalp
925.2	Crushing injury of neck
ICD-10	
S00.00XA	Unspecified superficial injury of scalp, initial encounter
S00.01XA	Abrasion of scalp, initial encounter
S00.03XA	Contusion of scalp, initial encounter
S00.10XA	Contusion of unspecified eyelid and periocular area, initial encounter
S00.11XA	Contusion of right eyelid and periocular area, initial encounter
S00.12XA	Contusion of left eyelid and periocular area, initial encounter
S00.201A	Unspecified superficial injury of right eyelid and periocular area, initial encounter
S00.202A	Unspecified superficial injury of left eyelid and periocular area, initial encounter
S00.209A	Unspecified superficial injury of unspecified eyelid and periocular area, initial encounter
S00.211A	Abrasion of right eyelid and periocular area, initial encounter
S00.212A	Abrasion of left eyelid and periocular area, initial encounter
S00.219A	Abrasion of unspecified eyelid and periocular area, initial encounter
S00.30XA	Unspecified superficial injury of nose, initial encounter
S00.31XA	Abrasion of nose, initial encounter
S00.33XA	Contusion of nose, initial encounter
S00.80XA	Unspecified superficial injury of other part of head, initial encounter
S00.81XA	Abrasion of other part of head, initial encounter
S00.83XA	Contusion of other part of head, initial encounter
S00.90XA	Unspecified superficial injury of unspecified part of head, initial encounter
S00.91XA	Abrasion of unspecified part of head, initial encounter
S00.93XA	Contusion of unspecified part of head, initial encounter
S01.00XA	Unspecified open wound of scalp, initial encounter
S01.01XA	Laceration without foreign body of scalp, initial encounter
S01.03XA	Puncture wound without foreign body of scalp, initial encounter
S01.101A	Unspecified open wound of right eyelid and periocular area, initial encounter
S01.102A	Unspecified open wound of left eyelid and periocular area, initial encounter
S01.109A	Unspecified open wound of unspecified eyelid and periocular area, initial encounter

© 2026 Wong GC et al. *JAMA Network Open*.

S01.111A	Laceration without foreign body of right eyelid and periocular area, initial encounter
S01.112A	Laceration without foreign body of left eyelid and periocular area, initial encounter
S01.119A	Laceration without foreign body of unspecified eyelid and periocular area, initial encounter
S01.131A	Puncture wound without foreign body of right eyelid and periocular area, initial encounter
S01.132A	Puncture wound without foreign body of left eyelid and periocular area, initial encounter
S01.139A	Puncture wound without foreign body of unspecified eyelid and periocular area, initial encounter
S01.141A	Puncture wound with foreign body of right eyelid and periocular area, initial encounter
S01.142A	Puncture wound with foreign body of left eyelid and periocular area, initial encounter
S01.149A	Puncture wound with foreign body of unspecified eyelid and periocular area, initial encounter
S01.20XA	Unspecified open wound of nose, initial encounter
S01.21XA	Laceration without foreign body of nose, initial encounter
S01.23XA	Puncture wound without foreign body of nose, initial encounter
S01.401A	Unspecified open wound of right cheek and temporomandibular area, initial encounter
S01.402A	Unspecified open wound of left cheek and temporomandibular area, initial encounter
S01.409A	Unspecified open wound of unspecified cheek and temporomandibular area, initial encounter
S01.411A	Laceration without foreign body of right cheek and temporomandibular area, initial encounter
S01.412A	Laceration without foreign body of left cheek and temporomandibular area, initial encounter
S01.419A	Laceration without foreign body of unspecified cheek and temporomandibular area, initial encounter
S01.421A	Laceration with foreign body of right cheek and temporomandibular area, initial encounter
S01.422A	Laceration with foreign body of left cheek and temporomandibular area, initial encounter
S01.429A	Laceration with foreign body of unspecified cheek and temporomandibular area, initial encounter
S01.431A	Puncture wound without foreign body of right cheek and temporomandibular area, initial encounter
S01.432A	Puncture wound without foreign body of left cheek and temporomandibular area, initial encounter
S01.439A	Puncture wound without foreign body of unspecified cheek and temporomandibular area, initial encounter
S01.80XA	Unspecified open wound of other part of head, initial encounter
S01.81XA	Laceration without foreign body of other part of head, initial encounter

S01.83XA	Puncture wound without foreign body of other part of head
S01.90XA	Unspecified open wound of unspecified part of head, initial encounter
S01.91XA	Laceration without foreign body of unspecified part of head, initial encounter
S01.93XA	Puncture wound without foreign body of unspecified part of head, initial encounter
S05.10XA	Contusion of eyeball and orbital tissues, unspecified eye, initial encounter
S05.11XA	Contusion of eyeball and orbital tissues, right eye, initial encounter
S05.12XA	Contusion of eyeball and orbital tissues, left eye, initial encounter
S05.20XA	Ocular laceration and rupture with prolapse or loss of intraocular tissue, unspecified eye, initial encounter
S05.21XA	Ocular laceration and rupture with prolapse or loss of intraocular tissue, right eye, initial encounter
S05.22XA	Ocular laceration and rupture with prolapse or loss of intraocular tissue, left eye, initial encounter
S05.30XA	Ocular laceration without prolapse or loss of intraocular tissue, unspecified eye, initial encounter
S05.31XA	Ocular laceration without prolapse or loss of intraocular tissue, right eye, initial encounter
S05.32XA	Ocular laceration without prolapse or loss of intraocular tissue, left eye, initial encounter
S05.60XA	Penetrating wound without foreign body of unspecified eyeball, initial encounter
S05.61XA	Penetrating wound without foreign body of right eyeball, initial encounter
S05.62XA	Penetrating wound without foreign body of left eyeball, initial encounter
S05.8X1A	Other injuries of right eye and orbit, initial encounter
S05.8X2A	Other injuries of left eye and orbit, initial encounter
S05.8X9A	Other injuries of unspecified eye and orbit, initial encounter
S05.90XA	Unspecified injury of unspecified eye and orbit, initial encounter
S05.91XA	Unspecified injury of right eye and orbit, initial encounter
S05.92XA	Unspecified injury of left eye and orbit, initial encounter
S07.0XXA	Crushing injury of face, initial encounter
S07.1XXA	Crushing injury of skull, initial encounter
S07.8XXA	Crushing injury of other parts of head, initial encounter
S07.9XXA	Crushing injury of head, part unspecified, initial encounter
S09.8XXA	Other specified injuries of head, initial encounter
S09.90XA	Unspecified injury of head, initial encounter
S09.92XA	Unspecified injury of nose, initial encounter
S09.93XA	Unspecified injury of face, initial encounter

© 2026 Wong GC et al. *JAMA Network Open*.

eTable 2. Maxillofacial Trauma Imaging *Current Procedural Terminology (CPT)* Codes

Code	Description
**Radiographs**	
Facial	
70140	Radiologic examination, facial bones; less than 3 views
70150	Radiologic examination, facial bones; complete, minimum of 3 views
Nasal	
70160	Radiologic examination, nasal bones, complete, minimum of 3 views
Orbit	
70200	Orbits 4 Views +
**Computed Tomography**	
70480	CT Orbit without contrast
70486	CT Maxillofacial without contrast

© 2026 Wong GC et al. *JAMA Network Open*.

eTable 3. Rates of Follow-up Computed Tomography Within 7 Days After Initial Plain Radiography

Imaging Type	Total No. of Patients	Follow-up CT Received, *n* (%)
XR	72125	3965 (5.5)
Facial	21100	1810 (8.6)
Nasal	42746	1530 (3.6)
Orbital	5048	350 (6.9)
Multiple	3231	275 (8.5)

© 2026 Wong GC et al. *JAMA Network Open*.

eTable 4. Diagnostic Outcomes After Initial Plain Radiography

Imaging Type	No. of patients with initial non-fracture diagnosis	New fracture diagnosis after imaging, *n* (%)	Delay >3 days, %
Plain Radiography	46166	11155 (24)	7.6
Facial	18233	1563 (8.6)	9.3
Nasal	21042	8718 (41)	7.2
Orbital	4796	179 (3.7)	13
Multiple	2095	695 (33)	7.8

© 2026 Wong GC et al. *JAMA Network Open*.

eFigure. Correlation matrix among all variables of interest (age, sex, region, urban status, location of the imaging episode, provider type of the imaging episode, insurance type, appropriate imaging indicator, year). Pearson correlation coefficients (ρ) were calculated for continuous-continuous variable pairs, Cramér's V for categorical-categorical pairs, and correlation ratios (η^2) for continuous-categorical pairs. The color intensity and displayed numbers indicate correlation strength, ranging from -1 to 1 for Pearson correlations, and 0 to 1 for Cramér's V and correlation ratios.

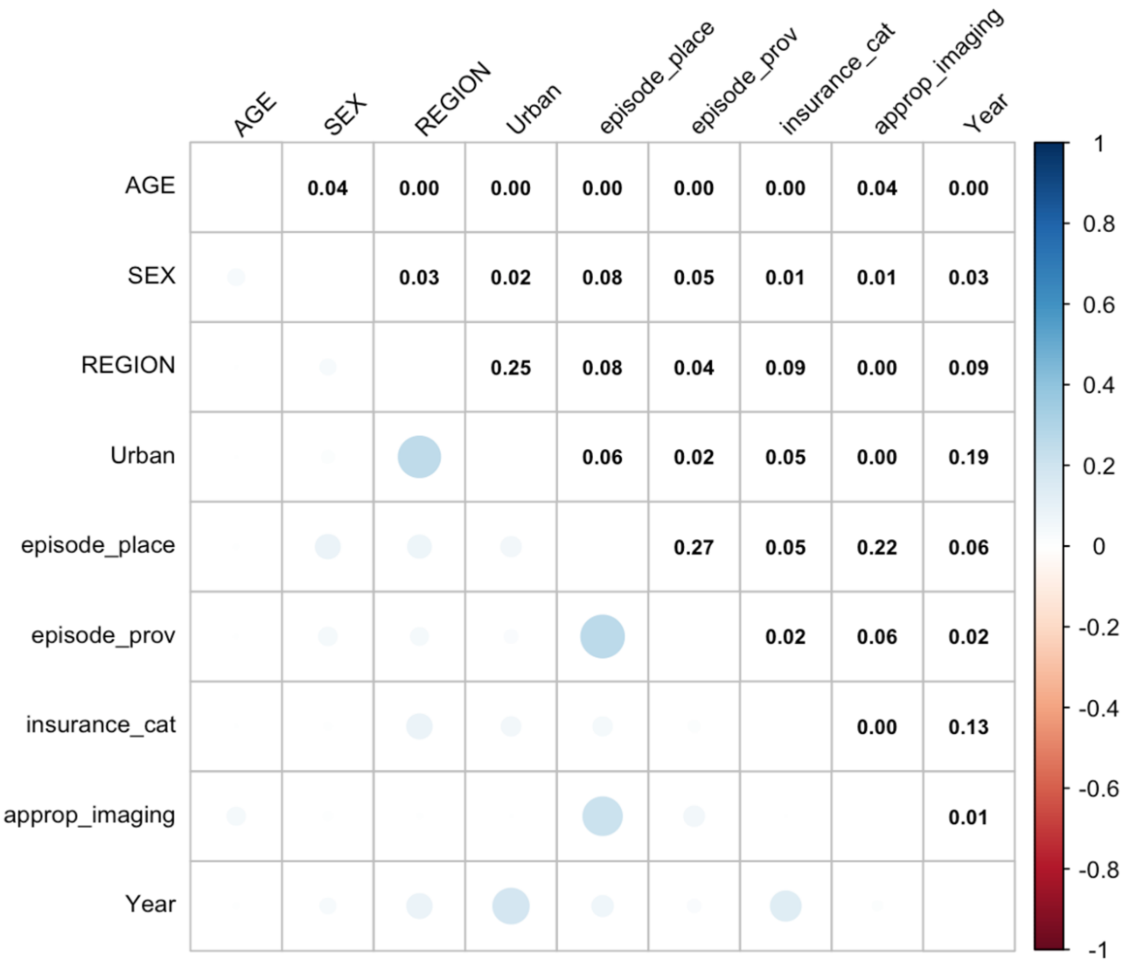

© 2026 Wong GC et al. *JAMA Network Open*.
