# Supplementary material for: Initial Imaging for Adults With Maxillofacial Trauma in a National Claims Database
Source: JAMA Netw Open. 2026 Feb 17;9(2):e2558293. doi: 10.1001/jamanetworkopen.2025.58293 (PMC12914484; doi:10.1001/jamanetworkopen.2025.58293)
Supplement: Supplement 2. — Data Sharing Statement [file jamanetwopen-e2558293-s002.pdf]

Data Sharing Statement

Wong. Initial Imaging for Adults With Maxillofacial Trauma in a National Claims Database. *JAMA Netw Open*. Published February 17, 2026. doi:10.1001/jamanetworkopen.2025.58293

Data
Data available: No

Additional Information
Explanation for why data not available: Data used was obtained from the Merative MarketScan database and is licensed for use by Merative.
